# The Effects of Cold Tolerance on the Distribution of Two Extreme Altitude Lizard Species in the Qinghai–Tibetan Plateau

**DOI:** 10.3390/ani15223297

**Published:** 2025-11-15

**Authors:** Xiaqiu Tao, Yiyi Li, Jiasheng Li

**Affiliations:** 1 Jiangsu Key Laboratory for Biodiversity and Biotechnology, College of Life Sciences, Nanjing Normal University, Nanjing 210023, China; 2 Department of Computer Science, New Mexico State University, Las Cruces, NM 88003, USA; gtarex@nmsu.edu; 3 College of Life Sciences, China Jiliang University, Hangzhou 310018, China

**Keywords:** temperature tolerance, prediction accuracy, species distribution models

## Abstract

Species distribution models (SDMs) have been widely used to predict potentially suitable habitats for species. However, traditional SDMs have been criticized for ignoring the physiological processes by which species respond to their environment. Integrating physiological tolerance into the model is essential to improve the prediction accuracy of SDMs. Currently, this approach has not been applied in the study of *Phrynocephalus erythrurus* and *Phrynocephalus theobaldi*, which are part of the world’s highest reptiles and endemic to Qinghai–Tibetan Plateau. In this study, based on experiments, we found that the critical thermal minimum (CT_min_) of the *Phrynocephalus theobaldi* was 0.85 °C. Further, we studied the effect of cold tolerance on the prediction of potential areas for these two reptile species. The high suitability area predicted by the SDMs incorporating cold tolerance data were 37.13% smaller than that predicted by the traditional SDMs. The difference between the two SDMs is primarily concentrated at the edges of the high suitability areas. The incorporation of cold tolerance data influenced the model’s predictions by effectively reducing the extent of edges of the high suitability areas. Our findings have theoretical significance for optimizing SDM predictions and provide a robust scientific basis for biodiversity conservation in the extreme-altitude ecosystems of the Qinghai–Tibetan Plateau.

## Simple Summary

Predicting where species occur is crucial for their research, but traditional models often overestimate suitable areas because they rely only on general climate. This is especially problematic for animals in extreme environments like the Qinghai–Tibetan Plateau, where surviving the harsh winter is critical. Our study aimed to improve distribution maps for two extreme altitude lizard species by adding a key piece of physiological information: their ability to withstand cold stress during winter hibernation. We compared maps based on just climate data with new maps that also incorporated this cold-tolerance trait. Our results showed that the new, physiology-informed models were smaller and more accurate, removing areas where winters are too severe for the lizards to survive. This proves that including an animal’s physiological limits in models is essential for realistic predictions. Creating more accurate habitat maps is valuable because it helps conservationists focus their efforts on the areas that are truly vital for protecting unique species in a changing world.

## 1. Introduction

Accurately defining the geographic distribution of species is a fundamental goal in ecology and conservation, providing the basis for effective management and biodiversity assessment [1]. Species distribution models (SDMs) have become indispensable tools for this purpose, correlating species occurrence records with environmental variables to predict habitat suitability across landscapes [2].

However, traditional SDMs often ignore biological traits such as physiological limits and dispersal capacity, leading to uncertainty in predictions [3,4]. Physiological traits significantly influence species’ climate vulnerability [5]. These traits often vary with elevations to adapt to cold temperatures, low oxygen, and high solar radiation at high elevations [5,6]. High-elevation ectotherms, for example, must tolerate extreme cold [7], but prolonged winter conditions may limit their survival and reproduction [8,9]. Incorporating cold-stress adaptations into models is thus critical for predicting climate impacts on these species.

As ectotherms, reptiles are highly sensitive to ambient temperature because body temperature governs physiology, behavior, and population dynamics [10,11]. Tropical ectotherms are more vulnerable to warming than temperate ones [12,13]. High-elevation species may benefit from warming due to broader thermal tolerances [14], but upward migrations could intensify competition [15]. Many lizards face extinction risks from climate change [16]. For example, recent work on Bedriaga’s rock lizards reports warming-driven physiological disruption and heightened risk [17]. Yet high-elevation species remain understudied [18,19].

The Qinghai–Tibetan Plateau, the highest and harshest plateau globally [20], offers unique insights into high-elevation species’ responses to climate change. With an average elevation exceeding 4000 m, the extreme diurnal temperature shifts, low oxygen, and intense UV radiation have shaped specialized physiological and ecological adaptations in plateau-dwelling lizards. Two toad-headed lizards, *Phrynocephalus theobaldi* Blyth, 1863 (IUCN: Least Concern [21]) and *Phrynocephalus erythrurus* Zugmayer, 1909 (IUCN: Least Concern [22]), occurring on the Qinghai–Tibetan Plateau, provide an excellent model system for exploring these questions. These two lizards belong to the viviparous *Phrynocephalus* radiation on the Qinghai–Tibet Plateau. Phylogeographic studies place *Phrynocephalus erythrurus* into two Qiangtang lineages, northern and southern, shaped by the Tanggula and Nyainqentanglha ranges and regional drainage [23]. Distributed across a vast and environmentally challenging landscape, these two lizards have evolved specific adaptations to cope with extreme temperatures and hypoxia [6]. In this study, we focus on two species with differing altitudinal ranges, *Phrynocephalus theobaldi* and *Phrynocephalus erythrurus*. We aim to investigate how incorporating a key physiological trait—cold stress experienced during the overwintering period—improves predictions of their current suitable habitats.

We developed two sets of models for each species: a traditional SDM using only bioclimatic and topographic variables, and a physiology-informed SDM that integrates a “cold stress frequency” variable. We hypothesize that the physiology-informed model will provide a more constrained and ecologically realistic estimate of suitable habitat. Specifically, we predict that the traditional model will overestimate the species’ ranges by failing to exclude areas where severe winter conditions exceed their physiological tolerance for cold. This study seeks to provide a more accurate understanding of the current distributions of these extreme altitude specialists and highlight the importance of integrating physiology into conservation biogeography.

## 2. Materials and Methods

### 2.1. Data Acquisition

**Figure 1:**
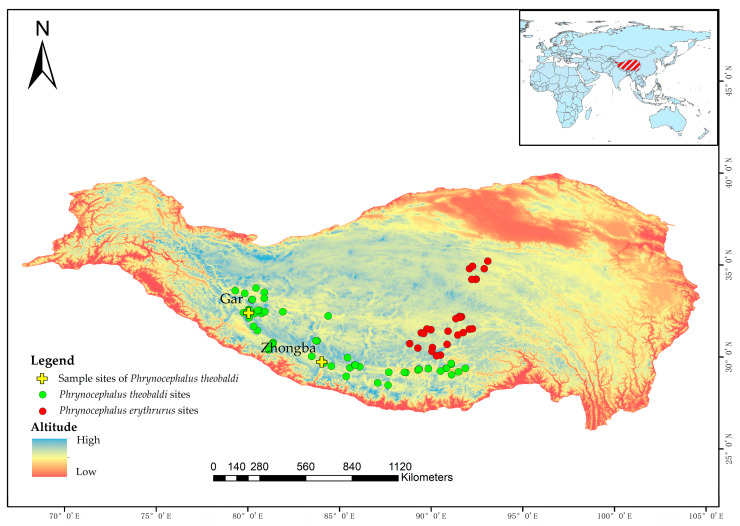
Presence data of two *Phrynocephalus* lizards used for SDMs generation, including CT_min_ sampling sites for *Phrynocephalus theobaldi*.

We compiled occurrence records of the two lizards from two sources: our own field observations (N = 62) and published literature (N = 34). In total, 96 raw records were collected. To reduce spatial autocorrelation, we applied the ENMTools package (v 1.1.5) [24] in R (v 4.3.1) [25] to filter records by retaining only one presence within each grid cell (30 arc-seconds, equal to 1 km), thereby ensuring spatial independence of the occurrence data. After spatial thinning, 86 occurrence points (*Phrynocephalus theobaldi*, 59; *Phrynocephalus erythrurus*, 27) were retained for subsequent analyses (Figure 1).

### 2.2. Predicting the CT_min_ and Cold Stress Frequency for Phrynocephalus

#### 2.2.1. Measuring the CT_min_ in *Phrynocephalus theobaldi*

The *Phrynocephalus theobaldi* used in the experiment were collected in July 2020 from Gar County (elevation: 4316 m.a.s.l.) and Zhongba County (elevation: 4561 m.a.s.l.) in Tibet. All individuals were adults with a snout-vent length greater than 40 mm.

The CT_min_ values were determined experimentally by placing individual lizards in a temperature-controlled chamber, where the temperature was lowered from 10 °C [26] at a rate of approximately 0.5 °C per minute. The CT_min_ was recorded as the temperature at which a lizard lost its righting response and could no longer perform voluntary movements (e.g., blinking, crawling), with the critical criterion that the animal fully recovered after being returned to ambient temperatures.

#### 2.2.2. Cold Stress Frequency During the Overwintering Period

We downloaded a dataset of 3-hourly soil temperatures for the year 2020 from the China Meteorological Data Service Center (https://data.cma.cn/, accessed on 10 October 2025). The dataset included measurements from 1950 national-level stations across the Qinghai–Tibet Plateau region of China, with a spatial resolution of 0.5°. We focused on soil temperatures at depths of 1–40 cm, as field investigations and previous literature indicate that most *Phrynocephalus* lizard nests on the Qinghai–Tibet Plateau are located at depths of approximately 30–50 cm [27,28]. Stations with incomplete data (i.e., missing temperature readings for any 3 h interval) were removed from the analysis. For each remaining station, soil temperature data were cropped to the overwintering period of the two lizard species, defined as 1 November to 31 March.

We defined a “cold stress event” as any 3 h interval in which the mean soil temperature (at 10–40 cm depth) fell below the critical thermal minimum (CT_min_). Besides *Phrynocephalus theobaldi*, the CT_min_ values for *Phrynocephalus erythrurus* used were −1.58 °C [29]. Correspondingly, a “cold stress frequency” was defined as a single day in which at least one cold stress event occurred. Subsequently, for each location, we calculated the “cold stress frequency” as the total number of cold stress days during the overwintering period (from 00:00 on 1 November to 23:00 on 31 March). This follows the event–day framework of Sun et al. [30] that was developed for heat stress. This variable was then used as a physiological predictor in our species distribution models.

### 2.3. Traditional Environmental Variables

We selected a suite of bioclimatic and topographic variables to model the habitat suitability for the two lizard species. 19 variables (version 2.1) were downloaded from the WorldClim database (www.worldclim.org, accessed on 10 October 2025) at a 30-arc-second (~1 km) resolution.

Topographic variables, including elevation, slope, and aspect, were derived from a 1 km resolution digital elevation model (DEM) obtained from OpenTopography (https://www.opentopography.org, accessed on 10 October 2025) to capture terrain-induced heterogeneity typical of high-mountain systems.

To avoid overfitting caused by multicollinearity, we conducted pairwise Spearman correlation analysis among all variables. When two variables were highly correlated (|r| ≥ 0.8), the variable with the higher contribution rate was selected. After this screening process, the predictor variables used for model construction are listed in Table 1.

### 2.4. Species Distribution Modelling

We predicted the potential distribution of lizards using BIOMOD2 (v 4.2-6-2) package [31] in R (v 4.3.1). Based on the number of occurrence records, 10 sets of 100 random pseudo-absence points were generated, which is considered sufficient for most machine-learning algorithms except for GLM and GAM [32]. 8 modelling algorithms were applied: classification tree analysis (CTA), generalized additive models (GAM), generalized boosted regression trees (GBM), generalized linear models (GLM), multivariate adaptive regression splines (MARS), maximum entropy (MaxEnt), random forests (RF), and extreme gradient boosting (XGBoost).

For model calibration, 80% of the occurrence data were used for training and 20% for testing. Cross-validation was repeated 10 times, resulting in a total of 1000 model runs. Model performance was assessed using three evaluation metrics: the area under the curve (AUC), the true skill statistic (TSS), and Cohen’s kappa. Only models that met the thresholds of TSS ≥ 0.8, AUC ≥ 0.7, and kappa ≥ 0.6 were retained for building the final ensemble projections [33,34].

## 3. Results

### 3.1. CT_min_ in Phrynocephalus theobaldi

The CT_min_ values used were 0.85 °C for *Phrynocephalus theobaldi*. There was no significant difference in the critical thermal minimum (CT_min_) of *Phrynocephalus theobaldi* between the two sampled populations (F_1,22_ = 1.816, *p* = 0.192) (Table 2). The variation in CT_min_ among individuals was also minimal, with an overall range of 0.7 °C (Table 2).

### 3.2. Model Performance and Evaluation

The 8 individual species distribution models constructed using the Biomod2 platform showed variable performance across 10 replicate runs. Evaluation metrics for these individual models ranged from 0.530 to 0.763 for KAPPA, 0.765 to 0.969 for AUC and 0.530 to 0.804 for TSS (Table 3).

By combining the outputs of the best-performing algorithms, the final ensemble models (EM) demonstrated excellent predictive power. In the traditional SDMs, the ensemble model for *Phrynocephalus theobaldi* achieved Kappa = 0.673, AUC = 0.978, and TSS = 0.877, whereas *Phrynocephalus erythrurus* reached Kappa = 0.706, AUC = 0.992, and TSS = 0.944. After integrating physiological predictors, TSS increased in both models. For *Phrynocephalus theobaldi*, ensemble model increased to Kappa = 0.705, AUC = 0.982, and TSS = 0.904. For *Phrynocephalus erythrurus*, the integrated ensemble yielded Kappa = 0.674, AUC = 0.989, and TSS = 0.965, indicating a high level of accuracy in predicting the distributions of both lizard species (Table 3). The final ensemble model for *Phrynocephalus theobaldi* integrated 300 individual models under the traditional framework and 420 models when incorporating CT_min_ data. For *Phrynocephalus erythrurus*, the ensemble model was composed of 323 traditional models and 342 physiology-informed models (Table 4).

### 3.3. Main Environmental Variables Among Different SDMs

**Figure 2:**
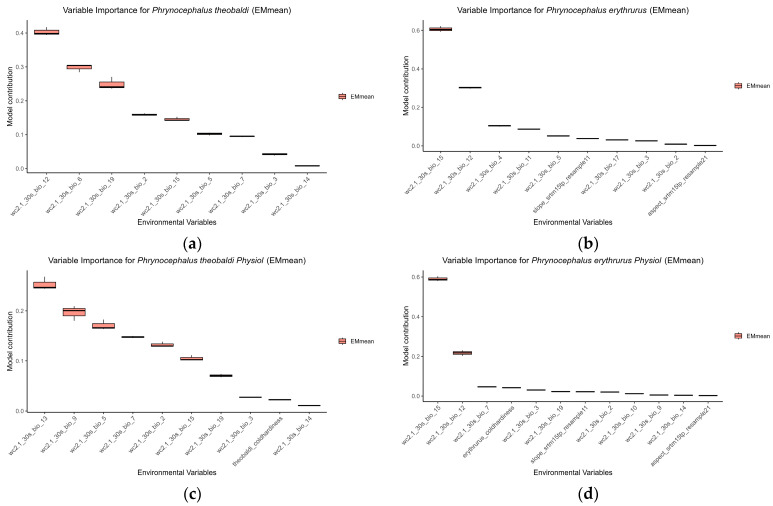
Mean contribution rates of sixteen environmental variables to the final ensemble traditional SDMs without incorporating CT_min_ data (**a**) *Phrynocephalus theobaldi* and (**b**) *Phrynocephalus erythrurus*, and with incorporating CT_min_ data (**c**) *Phrynocephalus theobaldi* and (**d**) *Phrynocephalus erythrurus*.

In the traditional SDMs (without CT_min_), the distribution of *Phrynocephalus theobaldi* was most influenced by six variables, each contributing over 10% to the final ensemble model: Bio12, Bio6, Bio19, Bio2, Bio15, Bio5, while Bio7 and Bio3 contributed over 5% (Figure 2a). For *Phrynocephalus erythrurus*, Bio15, Bio12 and Bio4 contributed more than 10%, while Bio11 and Bio5 contributed more than 5% (Figure 2b).

After incorporating the CT_min_ data, the set of influential predictors shifted. For the physiology-informed model of *Phrynocephalus theobaldi*, variables contributing over 10% became Bio13, Bio9, Bio5, Bio7, Bio2, Bio15, while Bio19 contributed over 5% (Figure 2c). For *Phrynocephalus erythrurus*, the model was simplified, with Bio15 and Bio12 remaining as the two variables that contributed more than 10% (Figure 2d).

In the traditional SDMs, the predicted suitability for *Phrynocephalus theobaldi* showed a decreasing trend with increasing Annual Precipitation (Bio12), but an increasing trend with higher Precipitation of the Coldest Quarter (Bio19) and Precipitation Seasonality (Bio15). Regarding temperature, suitability rose sharply once the Minimum Temperature of the Coldest Month (Bio6) increased above −20 °C, after which it gently declined. The optimal habitat corresponded to a Max Temperature of the Warmest Month (Bio5) of approximately 18–20 °C. The species also showed a gentle positive response to a larger Mean Diurnal Range (Bio2) (Figure S1).

For *Phrynocephalus erythrurus*, Precipitation Seasonality (Bio15) was the dominant factor, with suitability exhibiting a sudden jump when Bio15 approached a value of 110. Suitability also showed a positive trend with increasing Temperature Seasonality (Bio4), while the response to Annual Precipitation (Bio12) was relatively flat (Figure S2).

After incorporating CT_min_ data, both the key environmental factors and their response curves shifted. For *Phrynocephalus theobaldi*, suitability decreased with increasing Precipitation of the Wettest Month (Bio13). A significant increase in suitability was observed when the Mean Temperature of the Driest Quarter (Bio9) rose above −10 °C, after which the trend flattened. The response to Temperature Annual Range (Bio7) was complex, showing a slight dip around 30 °C before a sharp decline above 40 °C. For Mean Diurnal Range (Bio2), suitability was stable before rising markedly above a value of 12.5. The trends for Bio5 and Bio15 were consistent with the traditional model: suitability still peaked at a Max Temperature of the Warmest Month (Bio5) around 20 °C and increased with higher Precipitation Seasonality (Bio15) (Figure S3).

For *Phrynocephalus erythrurus*, the strong threshold for Precipitation Seasonality (Bio15) remained the primary factor, with Annual Precipitation (Bio12) as the secondary factor, although the peak suitability (Figure S4).

### 3.4. Effects of Incorporating CT_min_ on the Prediction of Suitable Habitat Areas

**Figure 3:**
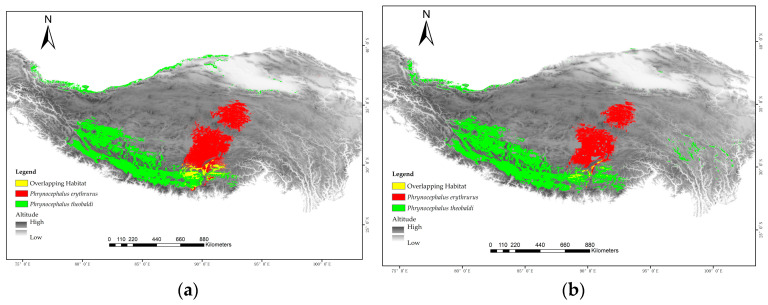
Prediction of suitable habitat for two lizards in Qinghai–Tibetan Plateau. (**a**) traditional SDMs, (**b**) physiology-informed SDMs.

**Figure 4:**
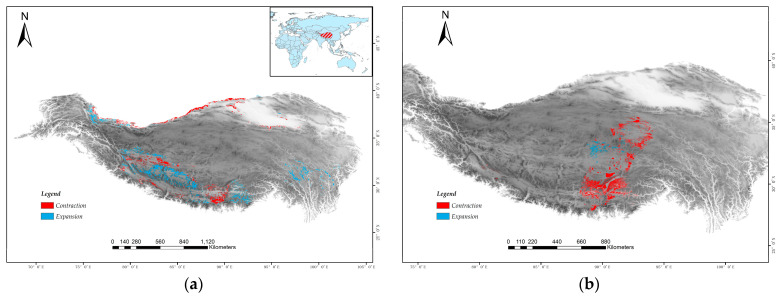
The difference between the two SDMs (with/without incorporating CT_min_ data) in predicting habitat of the (**a**) *Phrynocephalus theobaldi* and (**b**) *Phrynocephalus erythrurus* in Qinghai–Tibetan Plateau. Red areas indicate the reduction in suitable habitat after incorporating CT_min_ data, while blue areas indicate the expansion.

The prediction results of the two SDMs showed that the high suitability areas were mainly concentrated in South China, Central China, and East China, with a few in North and Southwest China, and almost no invasion risk in most of the northeast and northwest provinces of China (Figure 3). Although both SDMs performed well, the traditional model for *Phrynocephalus erythrurus* predicted a larger high suitability area (146,603 km^2^, Figure 3a) compared to the model incorporating CT_min_ data (106,906 km^2^, Figure 3b). Furthermore, the SDMs incorporating physiological data predicted a significantly smaller area (4865.97 km^2^, Figure 3a) of overlapping habitat between the two *Phrynocephalus* species, amounting to only 29.70% of the overlap projected by the traditional models (163,82.6 km^2^, Figure 3b). The two models show a difference of approximately 37.1% in high suitability areas for *Phrynocephalus erythrurus*, primarily concentrated at the edges of the high suitability areas, and the physiology-informed model projected a distribution that expanded further southward, rather than northward, and revealed several small additional patches that met the winter constraints (Figure 3 and Figure 4). These findings indicate that the incorporation of embryo temperature tolerance data influenced the model’s predictions by reducing the extent of edges within these high suitability areas.

## 4. Discussion

### 4.1. Comparison of CT_min_ Between Phrynocephalus theobaldi and Other Lizards in Qinghai–Tibetan Plateau

The critical thermal minimum (CT_min_) of an ectotherm is influenced by numerous factors, including developmental stage, geographical origin, and nutritional status [35]. In lizards, species inhabiting higher altitudes typically exhibit lower CT_min_ values. For example, *Phrynocephalus erythrurus*, found at the highest elevations, has a CT_min_ of approximately −1.58 °C [29], whereas *Phrynocephalus vlangalii*, which lives at relatively lower elevations, has a higher CT_min_ of 0.9 °C [36]. And the CT_min_ of the *Eremias argus* is approximately 1 °C [37], whereas the *Takydromus sexlineatus* and *Takydromus septentrionalis*, which live in lower latitude regions, is approximately 6 °C [38,39]. These interspecific differences likely reflect adaptations to the distinct thermal niches these species occupy. Our results showed that *Phrynocephalus theobaldi* possesses a CT_min_ intermediate to its high- and low-altitude congeners is consistent with the unique thermal environment it inhabits. This species is primarily distributed across open habitats in the southern Qinghai–Tibet Plateau, a region characterized by low annual mean temperatures (−5.1–8.3 °C) and a large diurnal temperature range (12–16 °C) [40]. The strong cold tolerance exhibited by *Phrynocephalus theobaldi* appears to be a key adaptation to these demanding thermal conditions.

Furthermore, the intermediate CT_min_ of *Phrynocephalus theobaldi*, which falls between that of higher- and lower-altitude congeners, indicates that tolerance is broader than in low-elevation specialists but narrower than in high-elevation specialists. This pattern implies a potential for localized niche and spatial overlap where suitable climatic and microhabitat conditions coincide, as has been noted in other lizard systems along elevational gradients [41]. Although our study did not directly test these hypotheses, the observed physiological variation underscores the role of cold tolerance in structuring species distributions on the Plateau.

### 4.2. Model Limitations and Behavioral Buffering

It is important to acknowledge the limitations of our modeling approach. Our predictions are based on soil temperature data at a 1 km resolution, which serves as a proxy for the thermal conditions within the lizards’ overwintering sites (hibernacula). However, this does not capture the microclimatic variations that individual animals experience. Ectotherms are known to engage in behavioral thermoregulation, and these lizards may actively select hibernacula with more favorable thermal profiles than the surrounding environment, a concept known as “behavioral buffering” [30]. For instance, lizards may choose deeper burrows or specific aspects of slopes that are better insulated from extreme cold. Studies on other lizard species have shown that females select specific nest sites to optimize thermal conditions for embryonic development (e.g., *Phrynocephalus przewalskii* [42], *Physignathus lesueurii* [43]). Previous studies indicate that repeated cold extremes or unusually long winters can overwhelm buffering capacities, leading to local extinctions despite apparently favorable microhabitat use [44].

### 4.3. Effects of Traditional Environmental Factors

In the traditional SDMs, three environmental factors, Bio2 (mean diurnal range), Bio3 (isothermality), and Bio15 (precipitation seasonality) were consistently retained across all four model combinations for both species. Notably, Bio15 contributed >10% in every ensemble model, indicating a strong signal of precipitation variability shaping habitat suitability on the Qinghai–Tibetan Plateau. Inspection of the response curves shows a marked increase in suitability with higher precipitation seasonality, implying that both *Phrynocephalus theobaldi* and *Phrynocephalus erythrurus* tend to occur in areas where rainfall variability is large. This pattern is ecologically plausible on the Qinghai–Tibetan Plateau, where the juxtaposition of Indian Summer Monsoon and mid-latitude westerlies produces pronounced seasonal and spatial gradients in precipitation and its variability [45]. In such environments, coarse sandy-gravel substrates and open steppe-desert vegetation co-vary with seasonal water availability, likely mediating prey and burrow microhabitats required by toad-headed lizards. Together, these results suggest that precipitation seasonality acts as a first-order filter for the two species, with thermal predictors (Bio2 and Bio3) refining the niche at finer scales.

Although elevation did not emerge as the main environmental variable across all sub-models after collinearity screening, recent SDM applications in mountainous fauna show that topography can substantially shape suitability patterns, fragmentation, and centroid shifts [46,47]. In parallel, taxonomic and phylogeographic work on *Phrynocephalus* lizards in Qinghai–Tibet Plateau indicates that the Plateau’s extreme topographic heterogeneity has promoted genetic differentiation and lineage/species formation [23,48,49]. Taken together, our study suggested that “elevation effects” on the both *Phrynocephalus theobaldi* and *Phrynocephalus erythrurus* are primarily mediated through temperature-related environments, as terrain can greatly influence factors such as temperature, hydrology, air pressure and solar radiation [50,51].

### 4.4. Effects of Incorporating Temperature Tolerance Data on the SDMs

Our study demonstrates that incorporating physiological data into SDMs significantly refines predictions of habitat suitability. A key finding is that the physiology-informed SDMs, which included CT_min_ data during overwintering, projected a smaller area of suitable habitat for *Phrynocephalus erythrurus* and distributional overlap between two lizards compared to the traditional climate-only model, which more accurately reflects the allopatric distribution of these two species observed in the field [27]. This better-predicting result aligns with a growing body of research suggesting that traditional SDMs often overestimate potential distributions because they do not account for critical physiological thresholds that can limit a species’ survival, even when climatic conditions appear favorable [52,53]. By failing to consider the lethal and sub-lethal effects of extreme cold events on overwintering lizards, traditional models may incorrectly identify large areas as suitable habitat. These eastern patches represent potential suitability rather than confirmed occurrences. They may reflect local relaxation of winter constraints, but they could also arise from sampling gaps or dispersal/historical barriers; moreover, year-round persistence may be limited by summer heat or aridity. We therefore recommend targeted surveys to evaluate these sites.

Similar findings have been reported in other taxa. For example, Laeseke et al. found that the distribution of *Capreolia implexa* was restricted by geographical barriers, causing climate-only SDMs to underestimate suitable areas [54]. Conversely, invasive species such as the red-eared slider (*Trachemys scripta elegans*) can disperse widely due to human activities, and climate-only SDMs often overestimate their potential range if they ignore physiological constraints [55]. When embryonic thermal limits were incorporated, unsuitable edge regions were excluded and high-risk areas were more realistically restricted to warmer provinces in southern and central China [56,57]. Likewise, Gamliel et al. reported that physiology-informed SDMs of marine organisms performed better than purely climatic models, especially at the edges of species’ ranges [4]. For intertidal crabs, adding experimentally determined thermal tolerance data reduced overly optimistic range predictions and provided a more conservative assessment of suitable habitats [58]. Collectively, these cases demonstrate that ignoring physiological bottlenecks can lead to both underestimation and overestimation of distribution ranges depending on species and context.

By integrating overwintering cold tolerance, our model effectively distinguishes between areas remain suitable through the overwintering period. For instance, a traditional model might identify a region as suitable based on warm summer temperatures, but our physiology-informed approach correctly excludes it if winter soil temperatures fall below the species’ lethal CT_min_. This refinement is particularly evident at the edges of the predicted distribution, providing a more ecologically realistic depiction of the species’ fundamental niche. More broadly, for high-mountain species the reliability of projections benefits from incorporating key, biology-linked and terrain-linked determinants (such as cold tolerance, preferred body temperature, thermal performance, stand metabolic rate and reproductive trait) [59,60,61], thereby reducing overprediction in climatically complex landscapes. This underscores the necessity of integrating key biological information to produce more reliable predictions [3,30].

## 5. Conclusions

Our study indicated that the embryo temperature tolerance data are important for the prediction of SDMs. We found that traditional species distribution models (SDMs) based on climatic and topographic variables overestimated the suitable habitat for both *Phrynocephalus theobaldi* and *Phrynocephalus erythrurus*, critically predicting a larger area of potential habitat overlap. By integrating CT_min_ data, the physiology-informed models corrected these inaccuracies, projecting smaller and more ecologically plausible habitats that align with the species’ known allopatric distributions. This research highlights that while broad climatic conditions may appear suitable, extreme physiological challenges, such as lethal winter temperatures, are a key limiting factor defining the true boundaries of species’ niches in extreme environments. In conclusion, our research provides a scientific basis for the conservation of these endemic species of the Qinghai–Tibetan Plateau. By providing more accurate habitat maps, our work can help guide targeted conservation strategies.

## Figures and Tables

**Table 1:** Predictor variables considered in potential distribution modeling.

Types	Variables	Description	*Phrynocephalus theobald*		*Phrynocephalus erythrurus*	
			Without CT_min_ Data	Incorporating CT_min_ Data	Without CT_min_ Data	Incorporating CT_min_ Data
Climatic variables	Bio1	Annual Mean Temperature				
	Bio2	Mean Diurnal Range (Mean of monthly (max temp–min temp))	✓	✓	✓	✓
	Bio3	Isothermality (Bio2/Bio7) (×100)	✓	✓	✓	✓
	Bio4	Temperature Seasonality (standard deviation × 100)			✓	
	Bio5	Max Temperature of Warmest Month	✓	✓	✓	
	Bio6	Min Temperature of Coldest Month	✓			
	Bio7	Temperature Annual Range (Bio5–Bio6)	✓	✓		✓
	Bio8	Mean Temperature of Wettest Quarter				
	Bio9	Mean Temperature of Driest Quarter		✓		✓
	Bio10	Mean Temperature of Warmest Quarter				✓
	Bio11	Mean Temperature of Coldest Quarter			✓	
	Bio12	Annual Precipitation	✓		✓	✓
	Bio13	Precipitation of Wettest Month		✓		
	Bio14	Precipitation of Driest Month	✓	✓		✓
	Bio15	Precipitation Seasonality (Coefficient of Variation)	✓	✓	✓	✓
	Bio16	Precipitation of Wettest Quarter				
	Bio17	Precipitation of Driest Quarter			✓	
	Bio18	Precipitation of Warmest Quarter				
	Bio19	Precipitation of Coldest Quarter	✓	✓		✓
Geographical factors	Altitude	Digital elevation model				
	Slope	Derived from DEM			✓	✓
	Aspect	Derived from DEM			✓	✓
Physiological factors	Cold stress frequency	total number of cold stress days during the overwintering period		✓		✓

✓ indicates that the variable was included in this model.

**Table 2:** Descriptive statistics for critical thermal minimum of *Phrynocephalus theobaldi*.

Population	N	Snout-Vent Length/mm	Critical Thermal Minimum/°C	Range
Gar	12	43.53 ± 1.58	0.9 ± 0.2	0.5–1.2
Zhongba	12	49.10 ± 3.62	0.8 ± 0.1	0.6–1.0

**Table 3:** Evaluation indices of species distribution model, expressed as mean ± SD.

	Species	*Phrynocephalus theobaldi*			*Phrynocephalus erythrurus*		
	Model	KAPPA	AUC	TSS	KAPPA	AUC	TSS
Traditional SDMs	CTA	0.530 ± 0.155	0.795 ± 0.086	0.557 ± 0.159	0.688 ± 0.147	0.904 ± 0.068	0.799 ± 0.144
	GAM	0.692 ± 0.125	0.924 ± 0.047	0.715 ± 0.114	0.566 ± 0.189	0.895 ± 0.073	0.650 ± 0.198
	GBM	0.669 ± 0.117	0.921 ± 0.043	0.681 ± 0.123	0.633 ± 0.198	0.954 ± 0.048	0.629 ± 0.217
	GLM	0.683 ± 0.130	0.893 ± 0.066	0.695 ± 0.130	0.533 ± 0.193	0.805 ± 0.109	0.600 ± 0.216
	MARS	0.704 ± 0.129	0.932 ± 0.054	0.708 ± 0.122	0.623 ± 0.196	0.938 ± 0.054	0.691 ± 0.199
	MAXENT	0.533 ± 0.161	0.765 ± 0.084	0.530 ± 0.167	0.587 ± 0.177	0.836 ± 0.097	0.672 ± 0.193
	RF	0.697 ± 0.122	0.936 ± 0.039	0.687 ± 0.130	0.615 ± 0.244	0.956 ± 0.040	0.575 ± 0.260
	XGBOOST	0.605 ± 0.165	0.877 ± 0.069	0.600 ± 0.169	0.629 ± 0.205	0.923 ± 0.085	0.622 ± 0.218
	EM *	0.673	0.978	0.877	0.706	0.992	0.944
SDMs With CT_min_ data	CTA	0.662 ± 0.159	0.870 ± 0.084	0.697 ± 0.152	0.707 ± 0.135	0.902 ± 0.061	0.803 ± 0.121
	GAM	0.705 ± 0.114	0.934 ± 0.038	0.720 ± 0.117	0.617 ± 0.195	0.916 ± 0.078	0.674 ± 0.217
	GBM	0.763 ± 0.113	0.948 ± 0.041	0.769 ± 0.115	0.707 ± 0.168	0.964 ± 0.034	0.722 ± 0.182
	GLM	0.681 ± 0.139	0.885 ± 0.073	0.694 ± 0.139	0.532 ± 0.202	0.814 ± 0.109	0.616 ± 0.225
	MARS	0.737 ± 0.122	0.932 ± 0.049	0.749 ± 0.121	0.654 ± 0.181	0.942 ± 0.046	0.727 ± 0.183
	MAXENT	0.603 ± 0.140	0.801 ± 0.074	0.602 ± 0.148	0.512 ± 0.188	0.798 ± 0.102	0.595 ± 0.203
	RF	0.757 ± 0.102	0.954 ± 0.027	0.757 ± 0.107	0.694 ± 0.200	0.969 ± 0.032	0.657 ± 0.220
	XGBOOST	0.656 ± 0.144	0.904 ± 0.056	0.652 ± 0.145	0.686 ± 0.181	0.925 ± 0.072	0.685 ± 0.199
	EM *	0.705	0.982	0.904	0.674	0.989	0.965

* Final ensemble model.

**Table 4:** Evaluation indices of species distribution model.

Species	*Phrynocephalus theobaldi*		*Phrynocephalus erythrurus*	
Model	Traditional SDMs	SDMs with CT_min_ Data	Traditional SDMs	SDMs with CT_min_ Data
CTA	18	54	72	67
GAM	59	56	37	34
GBM	44	68	38	53
GLM	49	50	26	30
MARS	48	66	40	45
MAXENT	15	21	36	23
RF	40	69	34	43
XGBOOST	27	36	40	47

## Data Availability

The data presented in this study are available on request from the corresponding author.

## Supplementary Materials

The following supporting information can be downloaded at: https://www.mdpi.com/article/10.3390/ani15223297/s1, Figure S1. Response curves of environmental variables in traditional SDMs for *Phrynocephalus theobaldi*. Variables are grouped by contribution to the model: (a) > 10 %; (b) ≥ 5 %; (c) < 5 %; Figure S2. Response curves of environmental variables in traditional SDMs for *Phrynocephalus erythrurus*. Variables are grouped by contribution to the model: (a) > 10 %; (b) ≥ 5 %; (c) < 5 %; Figure S3. Response curves of environmental variables in SDMs incorporating CT_min_ data for *Phrynocephalus theobaldi*. Variables are grouped by contribution to the model: (a) > 10 %; (b) ≥ 5 %; (c) < 5 %. Figure S4. Response curves of environmental variables in SDMs incorporating CT_min_ data for *Phrynocephalus erythrurus*. Variables are grouped by contribution to the model: (a) > 10 %; (b) < 5 %.
