# Supplementary material for: The Effects of Cold Tolerance on the Distribution of Two Extreme Altitude Lizard Species in the Qinghai–Tibetan Plateau
Source: Animals (Basel). 2025 Nov 15;15(22):3297. doi: 10.3390/ani15223297 (PMC12649369; doi:10.3390/ani15223297)
Supplement: Supplementary file 1 [file animals-15-03297-s001.zip › animals-3962430-supplementary.pdf]

Supplementary

(a) Model contribution > 10 %

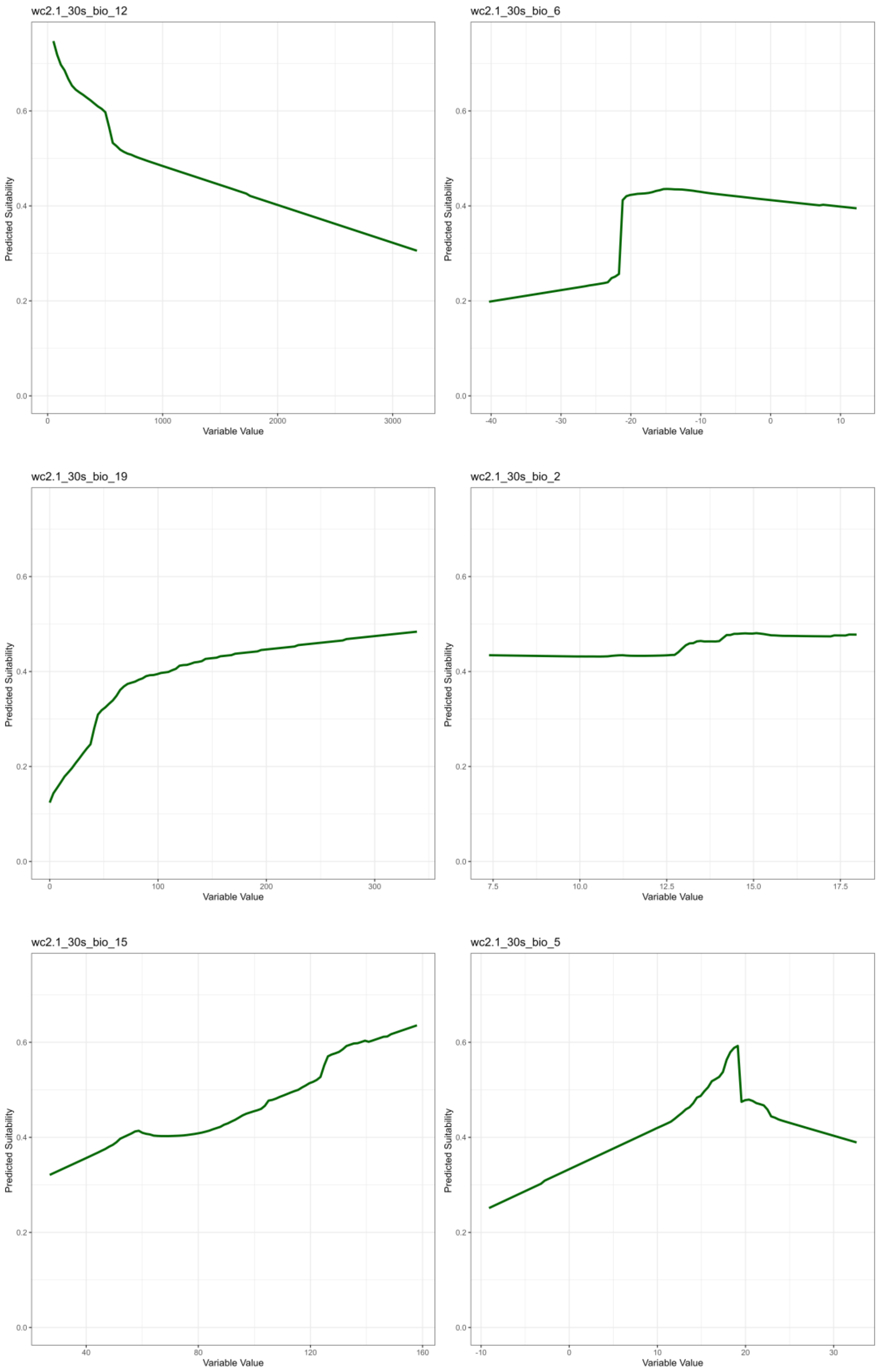

(b) Model contribution ≥ 5 %

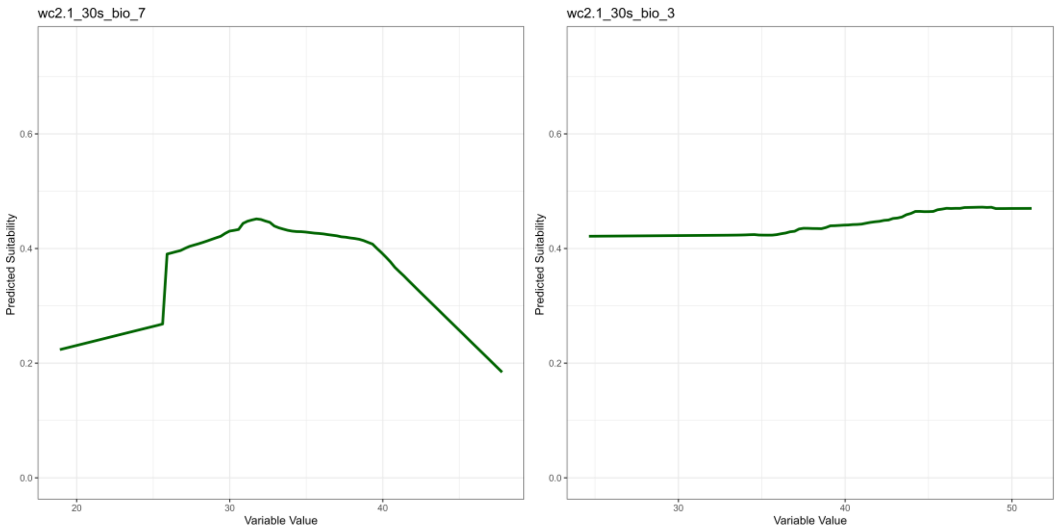

(c) Model contribution < 5 %

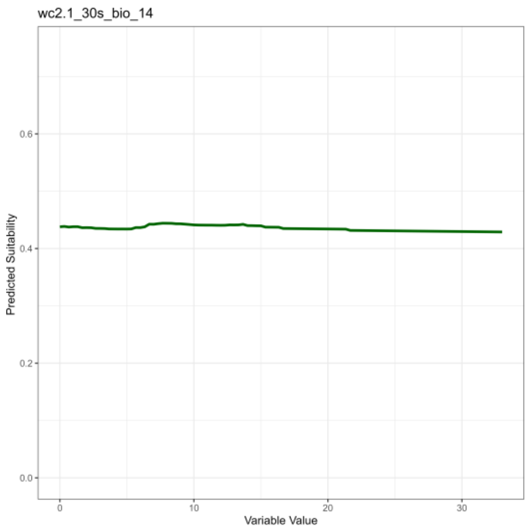

Figure S1. Response curves of the environmental variables of traditional SDMs in *Phrynocephalus theobaldi*. We divided them into three categories according to the contribution rate to the model, (a): variables of model contribution > 10 %; (b): variables of model contribution ≥ 5 %; (c): variables of model contribution < 5 %.

(a) Model contribution > 10 %

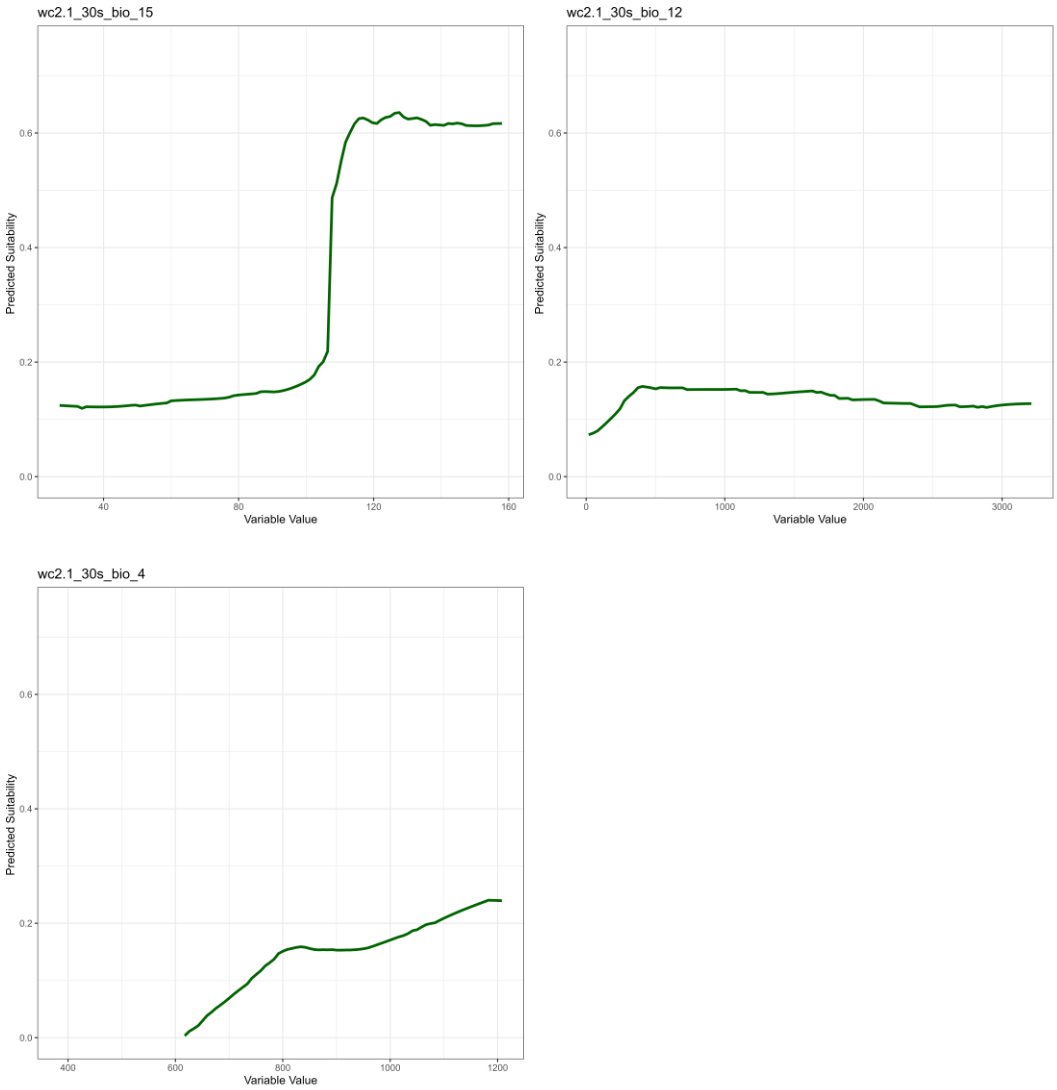

(b) Model contribution ≥ 5 %

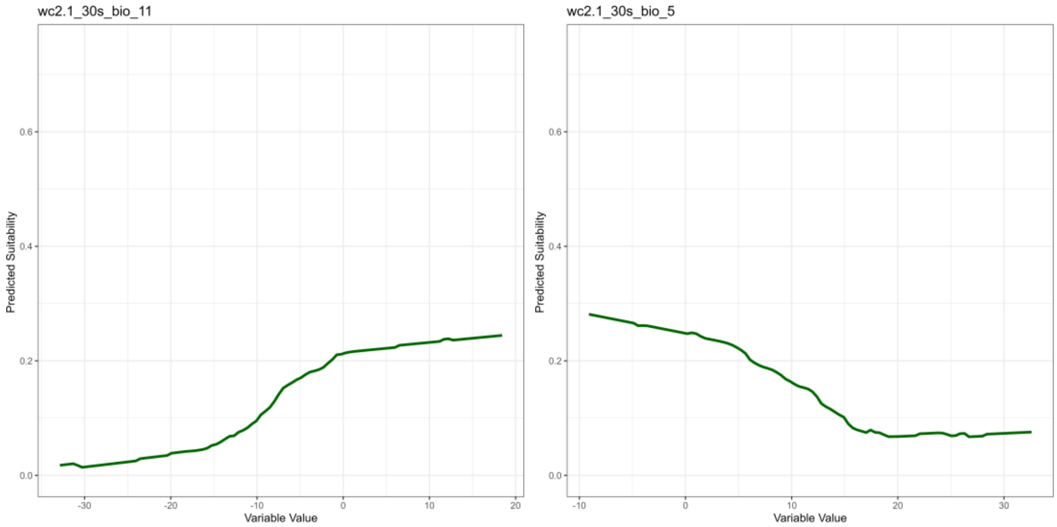

(c) Model contribution < 5 %

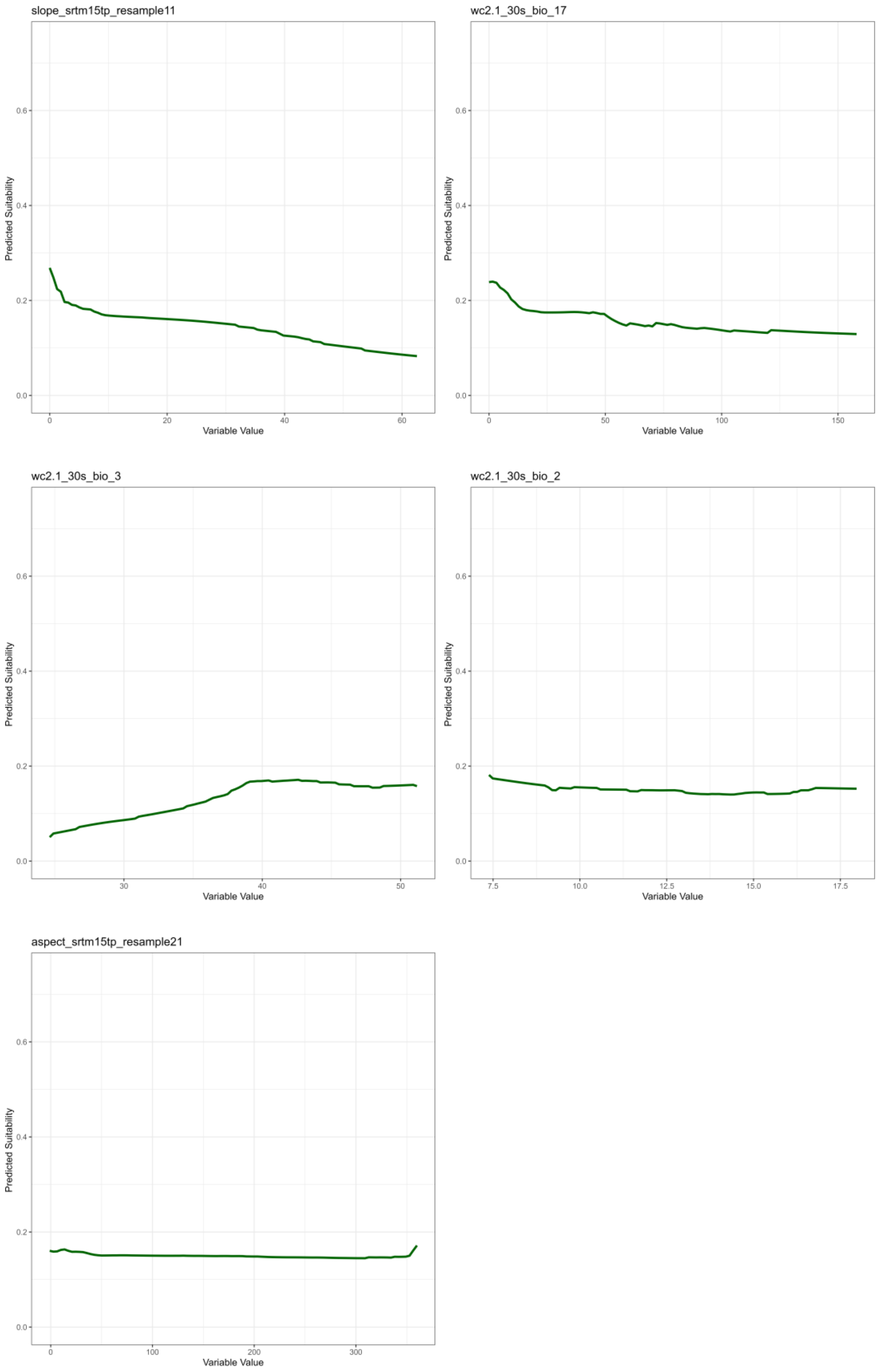

Figure S2. Response curves of the environmental variables of traditional SDMs in *Phrynocephalus erythrurus*. We divided them into three categories according to the contribution rate to the model, (a): variables of model contribution > 10 %; (b): variables of model contribution ≥ 5 %; (c): variables of model contribution < 5 %.

(a) Model contribution > 10 %

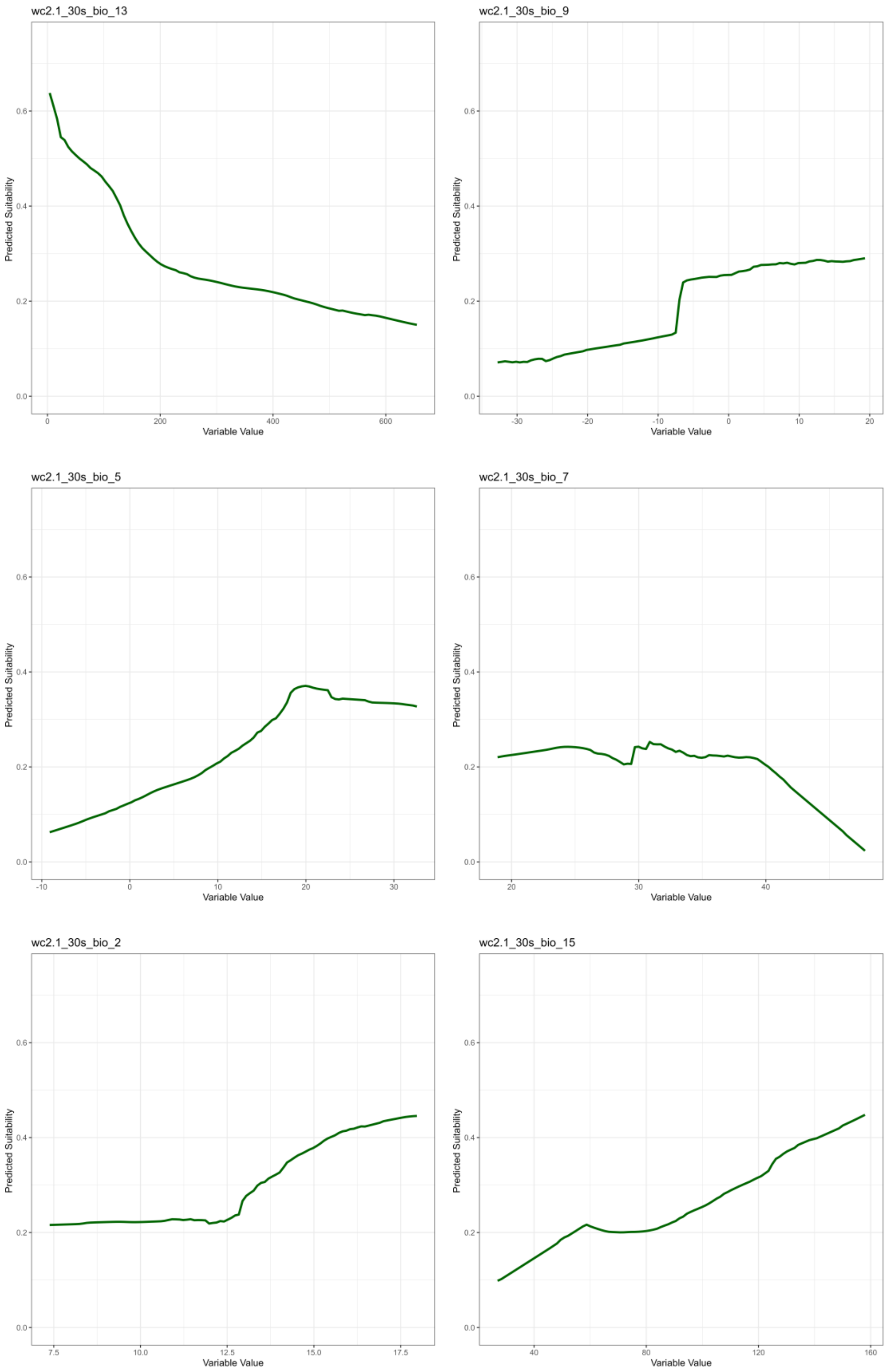

(b) Model contribution ≥ 5 %

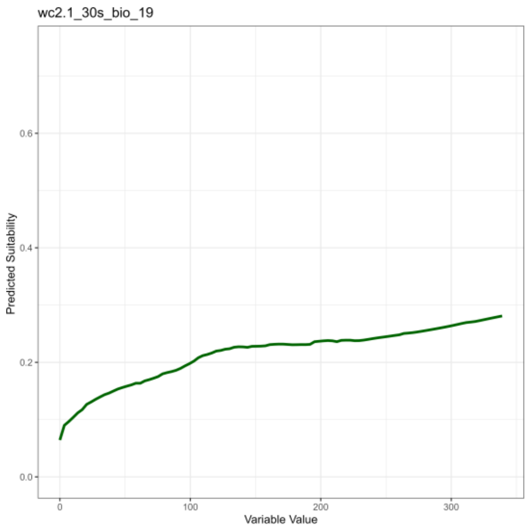

(c) Model contribution < 5 %

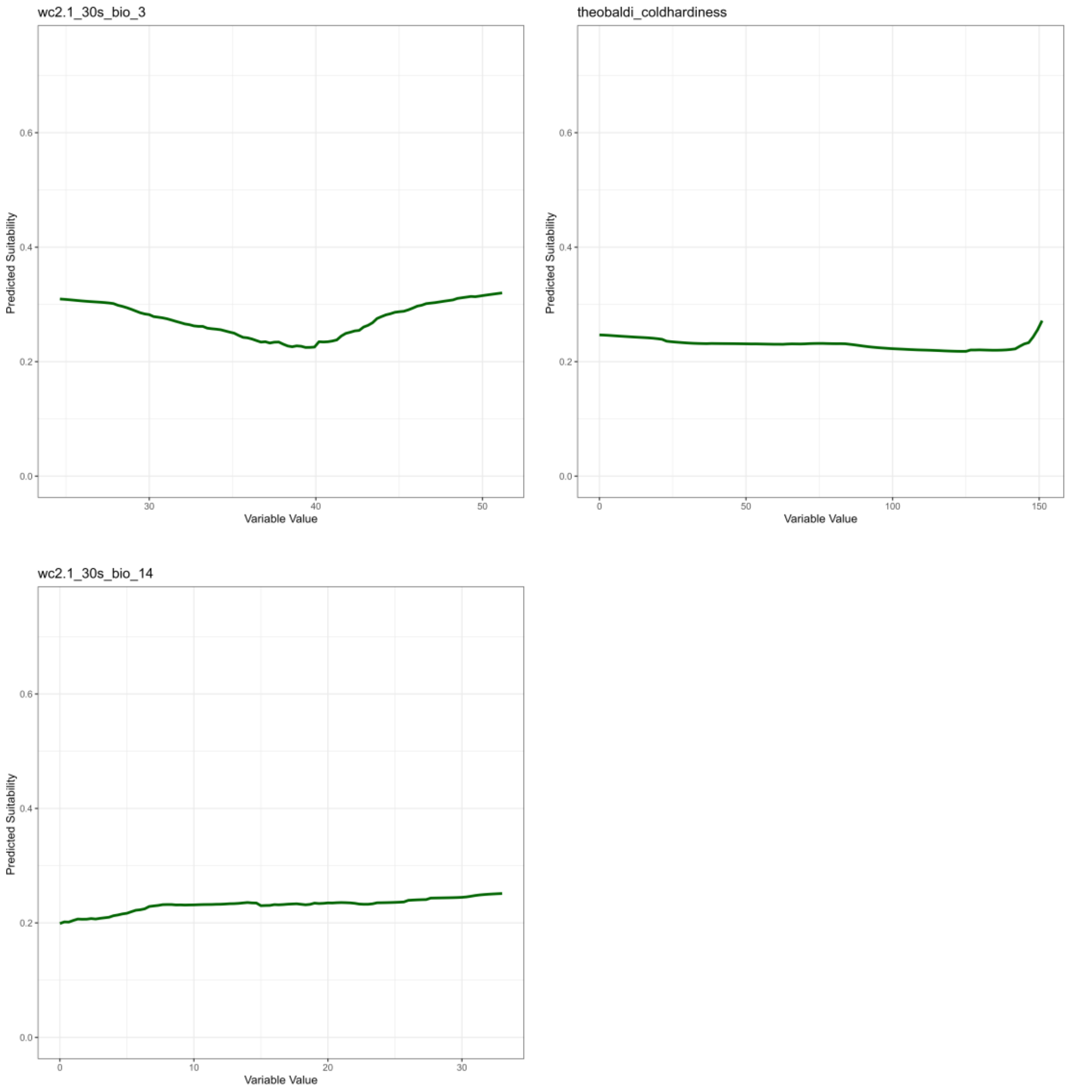

Figure S3. Response curves of the environmental variables of SDMs incorporating CT_{min} data in *Phrynocephalus theobaldi*. We divided them into three categories according to the contribution rate to the

model, (a): variables of model contribution > 10 %; (b): variables of model contribution ≥ 5 %; (c): variables of model contribution < 5 %.

(a) Model contribution > 10 %

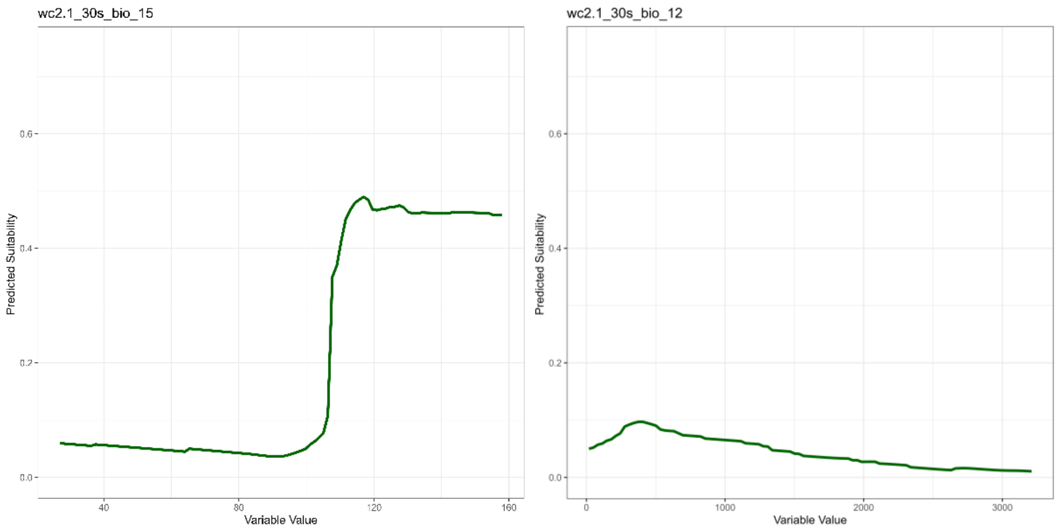

(b) Model contribution < 5 %

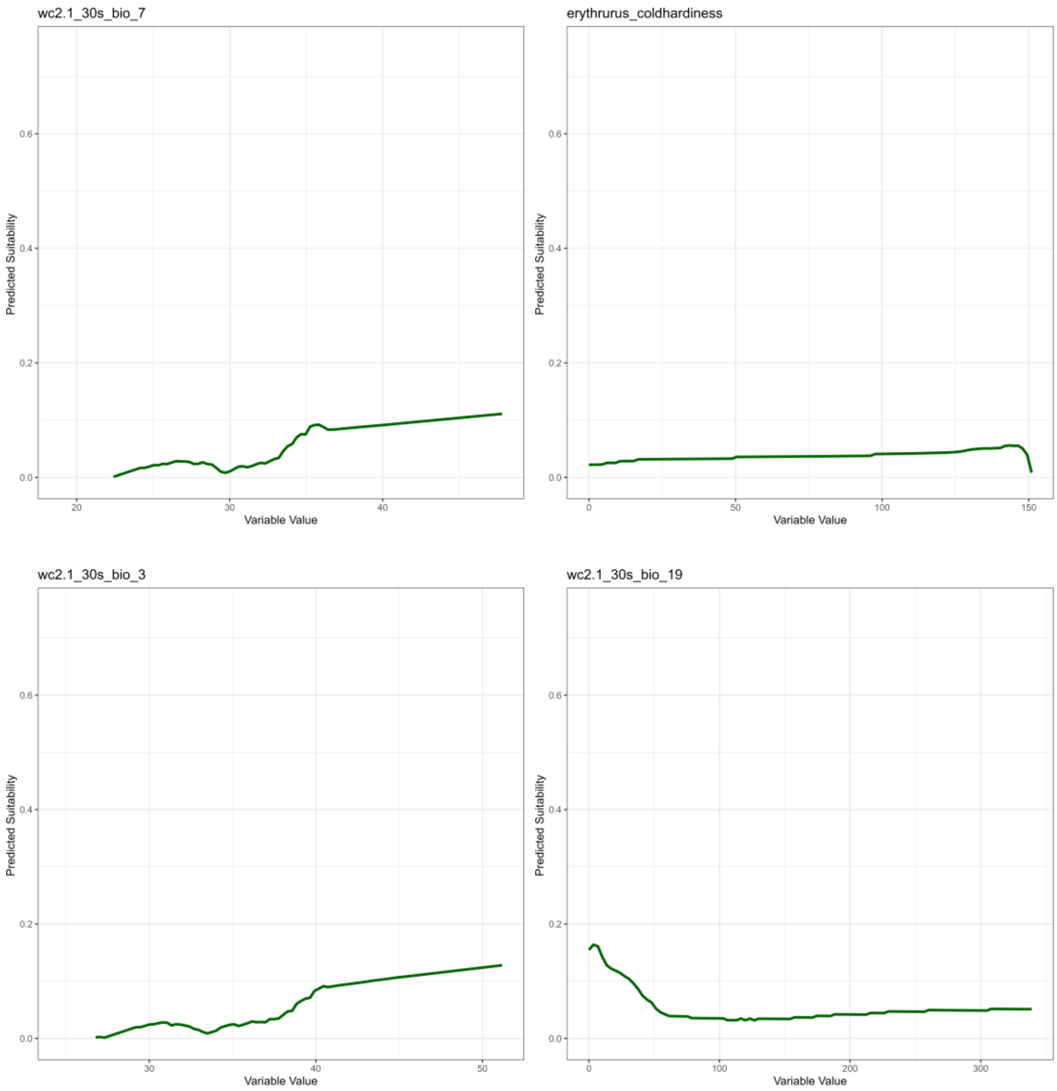

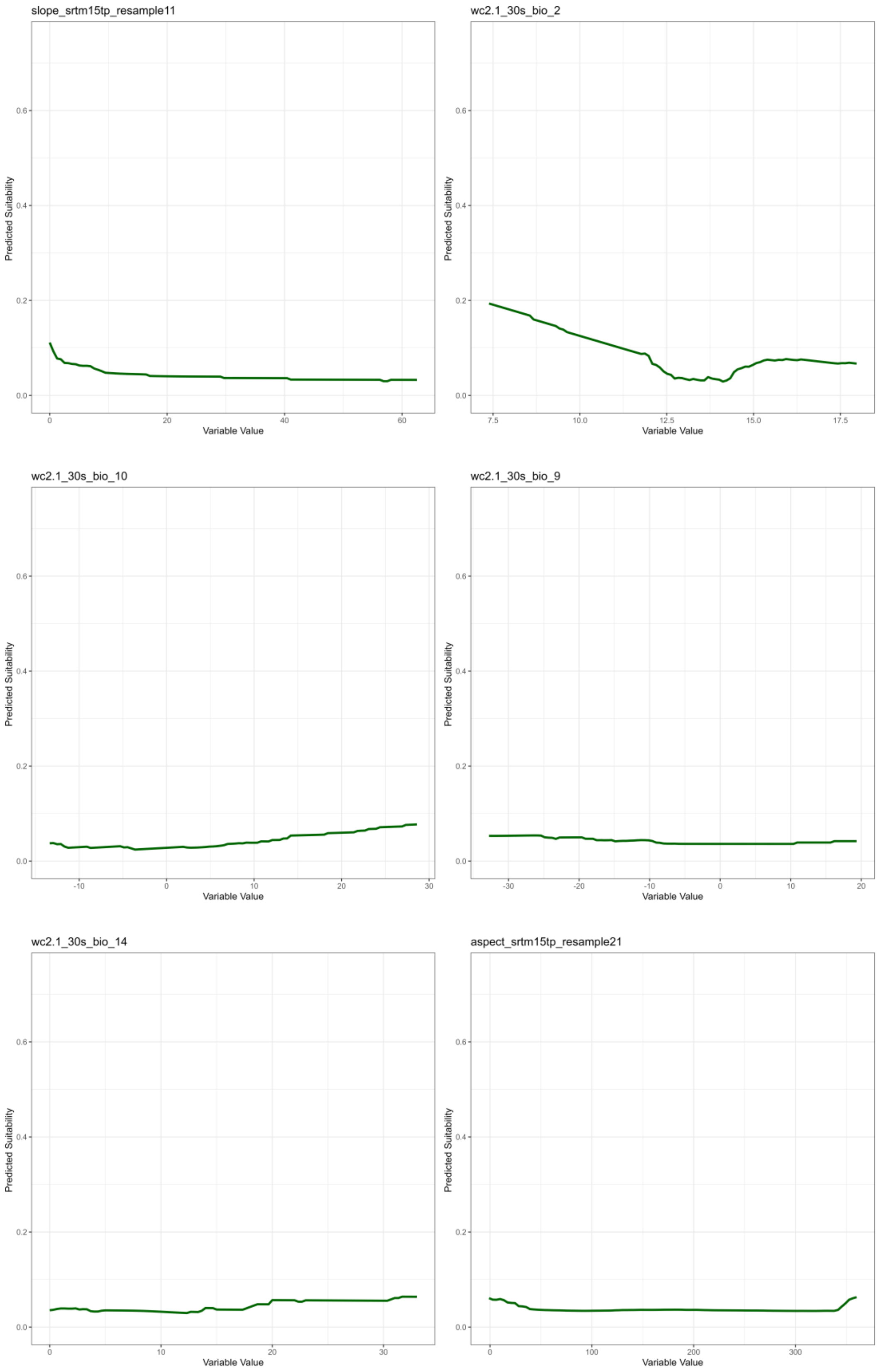

Figure S4. Response curves of the environmental variables of SDMs incorporating CT_{min} data in *Phrynocephalus erythrurus*. We divided them into three categories according to the contribution rate to the model, (a): variables of model contribution > 10 %; (b): variables of model contribution < 5 %.
